# SASBDB reaches 5000 data sets: empowering open science and next-generation SAS analysis

**DOI:** 10.1107/S2059798326000641

**Published:** 2026-02-03

**Authors:** Clement E. Blanchet, Aleksi Sutinen, Melissa A. Graewert, Dmytro Soloviov, Timur Tropin

**Affiliations:** a European Molecular Biology Laboratory (EMBL) Hamburg Germany; University of Cambridge, United Kingdom

**Keywords:** small-angle scattering, biological data banks, open data, method development

## Abstract

The Small-Angle Scattering Biological Data Bank (SASBDB) has recently reached a milestone of 5000 entries, reflecting over a decade of community-driven efforts to support open and reusable biological small-angle scattering data. SASBDB provides curated experimental scattering profiles together with metadata describing samples, experimental conditions and associated structural models, thereby enabling transparent data sharing, reproducibility and comparative analysis. The archive has become an important resource for benchmarking, reanalysis and method development, including the evaluation of structure-based modelling approaches and the integration of solution scattering data with high-resolution predictive models. Its growing content also supports benchmarking, method development, and emerging data-driven and machine-learning approaches that rely on curated collections of real experimental data. This milestone highlights the role of SASBDB as a foundational infrastructure for contemporary and future developments in biological small-angle scattering.

## Abstract 2

The Small-Angle Scattering Biological Data Bank (SASBDB) has surpassed 5000 deposited data sets, highlighting its role as a central open-access resource for biological small-angle scattering data and its growing impact on method development, benchmarking and data-driven analysis.

The Small-Angle Scattering Biological Data Bank (SASBDB; https://www.sasbdb.org) has recently passed 5000 deposited data sets, marking a significant milestone for the field. An overview of the growth and current composition of SASBDB is shown in Fig. 1 ▸. As a curated, open-access repository, SASBDB provides experimental small-angle scattering (SAS) data alongside comprehensive metadata including sample characteristics, measurement conditions, instrument settings, associated structural models and information on experimental provenance and contributor attribution. Over the past decade, it has evolved into an important resource for SAXS researchers, streamlining data deposition at publication and promoting transparency in peer review and data sharing. Beyond archiving, SASBDB increasingly supports structural model refinement and methodological innovation. This milestone not only expands the pool of real experimental data but also contributes to the development and evaluation of next-generation computational, including data-driven and machine-learning, methods at a time when the availability of high-resolution *AlphaFold* models positions SAS as a powerful tool for interpreting conformational states in solution at scale. Its growth reflects sustained contributions from the international BioSAS community, both in data deposition and in the development of reporting standards.

The establishment of SASBDB is rooted in a broader, community-driven effort that began in the mid-2000s to define publication standards and best practices for reporting biomolecular small-angle scattering experiments. These activities were shaped by extensive discussions within the SAS community, including the work of the IUCr Commission on Small-Angle Scattering and the Protein Data Bank SAS Validation Task Force, and resulted in the development and subsequent refinement of formal guidelines for data presentation, validation and transparency (Jacques *et al.*, 2012 ▸; Trewhella *et al.*, 2013 ▸, 2017 ▸). A key outcome of this process was the recommendation to create a dedicated public database for biological SAS data, together with a set of specific technical and scientific requirements for such an archive (Trewhella *et al.*, 2023 ▸).

Launched in 2014 (Valentini *et al.*, 2015 ▸), SASBDB provides a dedicated repository for solution scattering data from biological macromolecules (Fig. 1 ▸). Each data set includes the experimental one-dimensional scattering profile, accompanied by essential metadata: sample composition, construct sequence, buffer conditions, instrument settings, data-acquisition parameters and associated structural models and fits. The repository is curated, with consistency checks applied to metadata, profiles and model fits. Any detected anomalies are resolved in collaboration with the contributors to ensure completeness, internal consistency and transparent reporting of the data and the associated metadata for public reuse and peer review. Importantly, SASBDB does not impose acceptance or rejection criteria based on perceived data quality or fitness for a particular purpose; instead, in line with long-standing community guidelines, it aims to ensure that sufficient information is provided for users to assess the suitability of each data set for their specific scientific application (Jacques *et al.*, 2012 ▸; Trewhella *et al.*, 2017 ▸, 2023 ▸). Beyond cross-referencing to sequence and structure databases, SASBDB is now integrated into the federated ecosystem of structural biology archives through its coupling with the wwPDB and the PDB-IHM (Integrative/Hybrid Methods) framework. In this context, deposition of SAS data in SASBDB is a mandatory component of integrative structure depositions, ensuring that primary solution scattering data underlying hybrid models are archived, citable and reusable. This positions SASBDB as an early example of a domain-specific experimental archive operating as part of a broader, interoperable structural biology data infrastructure (Vallat, 2025 ▸). Although primarily focused on biomolecular form factors (proteins, nucleic acids, multi-domain systems and complexes), the archive has also begun to accommodate emerging sample types with less standardized metadata. With increasing interest in complex systems at the interface with soft-matter science, including lipid nanoparticles and related assemblies, SASBDB may provide a future destination for such data, provided that appropriate metadata standards continue to evolve. This inclusive approach balances openness with adherence to the adoption of open-data principles supporting findability and accessibility, ensuring broad utility for the scientific community.

A particularly notable contribution to SASBDB is the set of reference data sets generated through recent international SAXS comparison studies (Trewhella *et al.*, 2022 ▸), which provided high-quality scattering profiles measured across multiple facilities using standardized samples and protocols. These data sets now serve as essential benchmarks for evaluating data quality, reproducibility and the performance of modelling and validation methods. In parallel, several flexible or multi-domain systems deposited in SASBDB have been repeatedly reanalysed, serving as test cases for ensemble modelling and domain-arrangement refinement. Together, these examples demonstrate that SASBDB is not merely a repository but a shared framework for methodological evaluation and cross-laboratory collaboration within the biomolecular small-angle scattering community.

Beyond its role as an archive, SASBDB has become a central resource for method development and data-driven advances in small-angle scattering. Deposited data sets are widely reused for model refinement, validation of computational pipelines and the comparative evaluation of structure-based modelling and prediction methods (Brookes *et al.*, 2023 ▸; Trewhella *et al.*, 2024 ▸; Lytje & Pedersen, 2024 ▸; Ramirez *et al.*, 2025 ▸). As with many long-standing scientific data resources, large-scale computational studies typically rely on carefully curated subsets tailored to specific applications, reflecting the diversity of experimental conditions and historical deposition practices. Programmatic access to the archive is provided through a REST API, enabling the automated retrieval of data and metadata and supporting integration into computational workflows. This includes large-scale assessments of *AlphaFold*-predicted structures against experimental SAS data (Brookes *et al.*, 2023 ▸), as well as benchmarking of ensemble and domain-arrangement modelling strategies (Trewhella *et al.*, 2024 ▸). SASBDB profiles have also supported the development and validation of algorithms for molecular-weight estimation, curve description, explicit solvent scattering calculations and hybrid workflows that integrate atomic coordinates with SAS restraints (Lytje & Pedersen, 2024 ▸; Grant, 2018 ▸; de Oliveira Neto *et al.*, 2022 ▸; Hermann & Hub, 2020 ▸). The growing availability of high-resolution predictive models further expands the scope of SAS analysis, enabling systematic benchmarking and the development of data-driven approaches, and has begun to be used as a source of training and validation data for machine-learning methods, typically through carefully curated subsets tailored to specific applications. Recent methods, including *ab initio* density reconstruction, mixture deconvolution, coarse-grained modelling and ML-based interpretation, have already employed SASBDB data sets for training, validation or performance assessment (Grant, 2018 ▸; de Oliveira Neto *et al.*, 2022 ▸). This expanding reuse highlights the role of SASBDB as a foundational experimental resource for next-generation computational developments in SAS.

Beyond research reuse and method development, SASBDB has also become an excellent resource for educating the next generation of scattering practitioners by providing access to real experimental data together with rich metadata, profiles and associated models. Its publication-linked entries allow educators to seamlessly incorporate authentic data sets into course materials, helping to bridge the gap between classroom learning and real research practice. By working directly with these curated data sets, students can more readily transfer skills learned from textbook problems to the complexities of real scientific inquiry (Larsen *et al.*, 2025 ▸).

Looking ahead, the continued growth of SASBDB is expected to support increasingly sophisticated computational approaches for analysing solution scattering data. While the rate of deposition naturally reflects broader publication practices and experimental workflows, sustained community engagement remains essential to ensure continued expansion of the archive. As high-resolution predictive models become routinely available, the integration of SAS profiles with structural ensembles, conformational sampling and hybrid modelling workflows will place new demands on metadata completeness and standardization. At the same time, emerging machine-learning methods will require large, diverse and carefully annotated experimental data sets to ensure robust generalization across molecular systems and experimental conditions. Expanding support for SEC-SAXS, time-resolved measurements and nonstandard sample types will further broaden the scope of applications. Through sustained community engagement and continued evolution of deposition standards, SASBDB is well positioned to remain a central resource for transparent, reusable and data-driven SAS research.

## Figures and Tables

**Figure 1:**
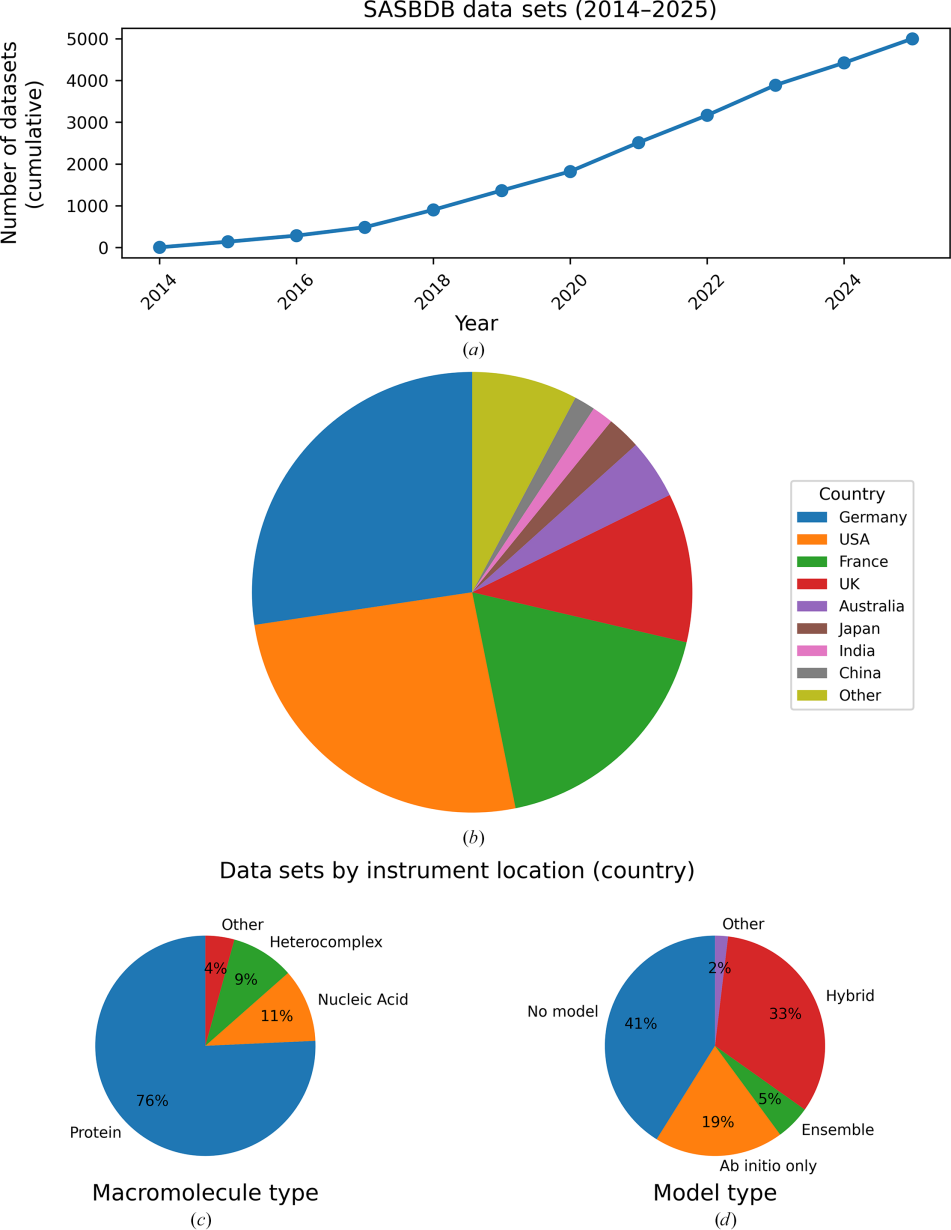
Overview of the SASBDB archive. (*a*) Number of data sets in SASBDB as a function of deposition year (cumulative count from 2014 to 2025). (*b*) Distribution of deposited data sets by country of the beamline or laboratory where data were collected. (*c*) Composition of the archive by macromolecule type. (*d*) Distribution of data sets by type of associated structural model.

## Data Availability

All data sets discussed in this article are publicly available through the Small-Angle Scattering Biological Data Bank (SASBDB; https://www.sasbdb.org). Information on the reuse of SASBDB data in published studies is maintained on the SASBDB website (https://www.sasbdb.org/reuse/).
